# The therapeutic efficacy of mesenchymal stem cells and their extracellular vesicles in the treatment of diverse skin diseases

**DOI:** 10.3389/fimmu.2025.1626066

**Published:** 2025-08-19

**Authors:** Mateusz Matwiejuk, Agnieszka Mikłosz, Hanna Myśliwiec, Adrian Chabowski, Iwona Flisiak

**Affiliations:** ^1^ Department of Dermatology and Venereology, Medical University of Bialystok, Bialystok, Poland; ^2^ Department of Physiology, Medical University of Bialystok, Bialystok, Poland

**Keywords:** extracellular vesicles, exosomes, mesenchymal stem cells, skin diseases, psoriasis, atopic dermatitis, systemic sclerosis, graft-versus-host disease

## Abstract

This review presents the current knowledge on the potential of mesenchymal stem cells (MSCs) and their extracellular vesicles (EVs) in the treatment of various skin diseases such as psoriasis, atopic dermatitis, contact dermatitis, systemic sclerosis, graft-versus-host disease, alopecia areata, and systemic lupus erythematosus. MSCs can modulate the immune response and release growth factors and cytokines that promote tissue regeneration and healing and reduce inflammation. In turn, EVs’ ability to transport various biological molecules, including microRNAs (miRNAs), makes them potential therapeutic agents. Moreover, EVs have been shown to reduce scaling, thickness, and erythema in psoriasis. In atopic dermatitis, EVs can alleviate clinical symptoms, lower serum IgE levels, and reduce immune cell infiltration. Mesenchymal stem cells like bone marrow-derived mesenchymal stem cells (BM-MSCs) exert their immunomodulatory effects by directly targeting various immune cell populations, including T cells, B cells, dendritic cells, and natural killer (NK) cells. To fully realize the potential of EVs in clinical practice, further research is needed to conduct well-designed clinical trials to evaluate the safety and efficacy of EV-based therapies in different skin diseases. Overall, EVs have the potential to revolutionize the treatment of skin diseases by offering a targeted and effective approach to address various underlying mechanisms, although further large-scale studies are needed.

## 1 Introduction

Psoriasis and atopic dermatitis (AD) are two autoimmune skin diseases with increasing prevalence.

Psoriasis is a long-lasting inflammatory skin disease that causes red, scaly patches on the skin. There is a significant genetic predisposition and autoimmune pathogenic traits. While psoriasis vulgaris, also called plaque-type psoriasis, is the most prevalent type, there are different clinical subtypes of psoriasis, each with its own dermatological manifestation. These subtypes include guttate, inverse, pustular, and erythrodermic (1). The classical clinical manifestations of psoriasis vulgaris are sharply defined borders of the affected skin areas, erythema, itchiness (pruritus), and silvery scales covering the affected areas. The plaques can grow and merge, covering large portions of the skin. The most common locations include the trunk, extensor surfaces of the limbs (elbows, knees) and scalp (2, 3). Inverse psoriasis is also known as flexural psoriasis and affects skin folds, or intertriginous areas, such as the groin, armpits, under the breasts, perianal region. Moreover, an inverse psoriasis is characterized by slightly eroded, red patches on the skin and an absence of the typical silvery scales seen in plaque psoriasis (4). Guttate psoriasis typically develops suddenly and often is triggered by a streptococcal infection. It is characterized by small, red, scaly patches that resemble drops of water. This is the most common type of psoriasis in children and adolescents. About one-third of individuals with guttate psoriasis may develop plaque psoriasis later in life (5, 6). Furthermore, pustular psoriasis is a type of psoriasis characterized by the formation of pus-filled blisters (pustules) on the skin. It can be localized or generalized. Localized pustular psoriasis is divided into psoriasis pustulosa palmoplantaris (PPP), which affects the palms and soles of the feet and is characterized by multiple, small, sterile pustules, and acrodermatitis continua of Hallopeau (ACH), which affects the distal parts of the fingers and toes as well as the nails. In turn, generalized pustular psoriasis is a severe and potentially life-threatening condition with rapid onset of widespread redness (erythema) and numerous pustules. It is often accompanied by systemic symptoms like fever, chills, and joint pain. It is important to note that pustular psoriasis can be a debilitating condition, and prompt medical attention is crucial for effective management (7). However, inflammation in psoriasis is not limited to the skin areas. It can affect various organs and systems, contributing to a range of comorbidities. Psoriatic arthritis is a common complication of psoriasis, affecting the joints and causing pain, swelling, and stiffness. Psoriasis has been linked to several other health conditions, including cardiovascular disease, metabolic syndrome, depression, and kidney disease (1).

While the most visible manifestations of psoriasis occur in the epidermis (outermost layer), the underlying dermis also plays a crucial role. The interaction between keratinocytes and various immune cells (for instance T cells and dendritic cells) within the dermis drives the inflammatory process (8). Dendritic cells (DCs) are indeed key players in the early stages of psoriasis, acting as professional antigen-presenting cells (APCs). Their activation in psoriasis is a complex process involving various factors, including antimicrobial peptides (AMPs) released by keratinocytes. In response to injury or inflammation, keratinocytes release AMPs like LL37, β-defensins, and S100 proteins (9)—for instance, LL37 acts as an antimicrobial peptide as well as signal that activates the immune system (10). Activated pDCs release type I interferons (IFN-α and IFN-β) that act on myeloid dendritic cells (mDCs). This leads to the maturation and activation of mDCs. Mature mDCs present antigens to T cells, leading to their activation and differentiation. Moreover, type I interferons promote the differentiation of T cells into Th1 and Th17 cells. Afterwards, Th1 cells produce IFN-γ, while Th17 cells release IL-17. Finally, IFN-γ and IL-17 are potent pro-inflammatory cytokines, and they stimulate keratinocytes to proliferate and differentiate abnormally, leading to the formation of psoriatic plaques (11–13).

Atopic dermatitis is a recurrent, chronic, non-infectious inflammatory skin disease (14). It commonly develops within the first year of life and affects over 200 million individuals worldwide. It is estimated that the total global prevalence of AD is around 3% to 4% in adults with female predominance and about 15%–25% in children (15). The disease is characterized by eczema-like rashes, erythema, papules, and exudative lesions, usually worsening at night. AD is classified based on age of onset, and the distribution of skin lesions changes as a person ages. AD is often categorized into infantile, childhood, adolescent, and adult subtypes. In infants (usually aged 0–2 years), lesions often affect the face (cheeks, forehead), scalp, and extensor surfaces of the limbs (outer elbows and knees), but the diaper area is usually spared. In patients aged between 2 and 12 years, cutaneous manifestations often transfer to the flexural surfaces (antecubital fossa, popliteal fossa), wrists, ankles, and neck. In adult patients with AD (usually 12+ years), cutaneous lesions occur with local or widespread involvement (16). Atopic dermatitis is a member of a triad called the “atopic triad,” along with allergic rhinitis and bronchial asthma. These conditions often occur together in individuals, either simultaneously or sequentially, and are linked by a shared predisposition and immune response. In patients with atopic dermatitis, the overall prevalence of rhinitis is 40.5%, asthma 25.7%, or both at 14.2%. On the other side, AD may be associated with non-atopic comorbidities such as autoimmune, cardiovascular, endocrine, infectious, ocular, and psychiatric diseases and some cancers (breast, brain, keratinocyte, lung, pancreatic cancer, lymphoma, and melanoma) (17). The pathogenesis of AD is complex and combines skin barrier dysfunction, dysregulation of innate and adaptive immune system, dysbiosis of the skin bacterial microbiome, genetic predisposition, and environmental factors. It is believed that the epidermal barrier defect is associated with mutations in the filaggrin gene, which, in combination with ceramide deficiency, lead to transepidermal water loss and increased penetration of irritants, allergens, and microorganisms into the skin. In turn, the disruption of the skin barrier leads to a cascade of events, including chronic inflammation, epidermal hyperplasia, and the infiltration of immune cells. These immune cells include dendritic cells, eosinophils, and T lymphocytes, all contributing to the inflammatory response. Dysregulation of a type-2 T-helper cell (Th2), Th17, and Th22 inflammatory response plays a crucial role in the pathogenesis of the disease. As a result, a broad range of pro-inflammatory cytokines like interleukins IL-2, IL-4, IL-5, IL-12, IL-13, IL-18, IL-21, and IL31, tumor necrosis factor α (TNF-α), and interferon γ (IFN-γ) are produced. These cytokines contribute to skin barrier dysfunction, inflammation, and disease progression (18).

One promising approach for treating inflammatory skin diseases involves the use of mesenchymal stem cells (MSCs). MSCs have demonstrated the ability to modulate the immune system, repair damaged tissues, and stimulate angiogenesis (19). It has been provided a strong support for the therapeutic efficacy and safety of intravenously injected bone marrow-derived mesenchymal stem cells (BM-MSCs) in various inflammatory skin conditions—for example, in acute and chronic graft-versus-host disease (GvHD) with skin manifestations (20), systemic lupus erythematosus (SLE) (21), and systemic sclerosis (SSc) (22). The beneficial effects of MSCs’ applications in tissue repair are attributed to their paracrine action via the secretion of vesicles such as extracellular vesicles (EVs) rather than to cell engraftment and response to the site of injury. Several studies indicate that MSC-derived EVs represent an alternative to MSC transplantation and can even replace stem cell-based therapy. Indeed MSC-derived EV therapy has been successfully used in various disease models, including diabetes, cancer, skin injuries, neurological, cardiovascular, immunological, renal disorders. While EV therapy holds promise, potential risks need careful consideration. Though generally less immunogenic than whole cells, EVs can still trigger immune responses or interact negatively with the host’s immune system, especially at high doses or with prolonged use. This could affect hemostasis, potentially leading to thrombosis. Furthermore, the variability in EV production and isolation methods can impact consistency and therapeutic effectiveness, posing challenges for standardization and large-scale manufacturing.

Extracellular vesicles (EVs), naturally secreted by cells, have recently emerged as pathophysiological mediators of psoriasis. EVs come in various sizes, ranging from smaller exosomes (30–150 nm) to larger microvesicles (100–1,000 nm) and apoptotic bodies (1,000–5,000 nm) (12). Furthermore, EVs play a prominent role in the modulation of several biological processes. Firstly, EVs can attenuate inflammation by increasing the secretion of anti-inflammatory cytokines and promoting Treg polarization. They can also transport antigens and co-stimulatory molecules, contributing to immune cell memory and tolerance (23). Secondly, EVs can be involved in host defense against pathogens. They can contain antimicrobial factors like peptides and proteins, and they can also promote immune responses that help eliminate bacteria (24). Thirdly, EVs can influence cell growth and movement by delivering growth factors, signaling molecules, and extracellular matrix components. This is particularly important in processes like wound healing and development (25). Finally, EVs can stimulate the formation of new blood vessels, a process known as angiogenesis. This is essential for tissue growth and repair, but it can also contribute to pathological conditions like cancer (26, 27).

MSC-derived extracellular vesicles (EVs) universally suppress Th1/Th2/Th17 axis across different skin diseases such as psoriasis and atopic dermatitis. Psoriasis and AD are both inflammatory skin conditions, but their underlying immune mechanisms differ. Psoriasis is primarily driven by the Th17/IL-17 pathway, while AD involves multiple pathways, including Th2, Th22, and potentially Th17. This difference in immune involvement explains why targeting the Th17/IL-17 axis is highly effective for psoriasis, but a broader approach targeting multiple pathways is needed for AD. EVs can modulate immune responses, and their effects on different T helper cell subsets are distinct. For Th1 cells, EVs typically reduce the levels of pro-inflammatory cytokines like IFN-γ, TNF-α, and IL-2 (28). Conversely, for Th17 cells, the immunomodulatory effect of EVs is often characterized by a reduction in the levels of IL-17, IL-21, and IL-22. Both IL-17 and IL-22 drive keratinocyte proliferation and contribute to the inflammatory environment characteristic of psoriasis (29). Therefore, keratinocytes, the main cells of the epidermis, are a primary target of EVs in psoriasis. Mast cells are the target for EVs in AD. They contribute to inflammation not only through immediate hypersensitivity reactions but also by releasing a variety of cytokines and chemokines that further amplify the inflammatory response (29). This differential impact on cytokine profiles suggests that EVs can selectively influence the activity of these T helper cell subsets. MSC-derived EVs transfer miRNAs that suppress T-cell proliferation and cytokine production, inhibiting the differentiation of pro-inflammatory Th1 and Th17 cells and promoting the generation of regulatory T (Treg) cells. Th1 and Th17 cell differentiation is regulated by specific cytokines and transcription factors. Th1 cells are driven by IL-12 and T-bet via the STAT4 pathway, whereas Th17 cells are promoted by TGF-β, IL-6, and IL-23, engaging the RORγt and STAT3 pathways. EVs can modulate these pathways by delivering molecules like miRNA, proteins that inhibit the expression of key transcription factors such as T-bet and RORγt, thereby reducing the IFN-γ and IL-17 levels (30). In addition, EVs can also promote a shift in macrophage phenotype from pro-inflammatory (M1) to anti-inflammatory (M2) states, which are beneficial for tissue repair and inflammation resolution (30).

Extracellular vesicles are excellent candidates for the treatment of chronic inflammatory skin diseases due to their favorable biological properties such as biocompatibility, stability, and low toxicity. These small vesicles are involved in the intercellular transportation of biologically active molecules including nucleic acids, transcription factors, cytokines, carbohydrates, lipids, proteins, extracellular matrix proteins, receptors, and antigens. The great potential of the EVs is used for controlled drug delivery, gene delivery (due to their microRNA and mRNA content), and biomarker-driven therapies. Despite the innate benefits of MSC-derived EVs, the cell-free therapy has some drawbacks, including low targeting efficiency, low yield, limited tissue repair capabilities, and limited drug delivery capabilities. Therefore, some genetic modifications or pretreatment of EVs are proposed to increase the effectiveness of the cell-free strategy.

In recent years, researchers have mainly focused on different aspects of the use of MSC-derived EVs in the therapy of many diseases. While the exact implementation in dermatoses still remains not completely explained, the application of these EVs in skin conditions is an area of proceeding research. This narrative review aims to evaluate the potential implementation of EVs in widespread skin diseases such as psoriasis, atopic dermatitis, contact dermatitis, systemic sclerosis, graft-versus-host disease, alopecia areata, and systemic lupus erythematosus.

## 2 Discussion

### 2.1 Mesenchymal stem cell-derived extracellular vesicles

#### 2.1.1 Psoriasis

Bakr et al. (31) investigated the therapeutic potential of BM-MSCs and their derived EVs (i.e., exosomes) in psoriasis-like inflammatory changes in animal models. The researchers used a psoriasis model induced by imiquimod (IMQ) in female albino rats. The rats were divided into four groups: control, IMQ-only, IMQ + BM-MSCs, and IMQ + BM-MSC-derived exosomes. The results showed that both BM-MSCs and exosomes reduced the inflammatory changes in the skin as observed through general observations and a microscopic examination of stained sections. Additionally, it was found that BM-MSCs secrete various cytokines that shift T lymphocyte populations from effector T cells to regulatory T cells. These cytokines include transforming growth factor-beta (TGF-β), hepatocyte growth factor (HGF), prostaglandin E2 (PGE2), nitric oxide (NO), and indoleamine 2,3-dioxygenase (IDO). Finally, BM-MSCs can directly interact with NK cells through cell–cell contact. This interaction leads to the downregulation of activating receptors on NK cells. Consequently, the cytotoxic activity of NK cells was reduced, leading to an immunosuppressive effect. Furthermore, BM-MSCs secrete various paracrine factors, such as IL-6, PGE2, and GRO-γ, which can suppress the maturation of dendritic cells (DCs). Additionally, BM-MSCs can interact with DCs through Jagged-2, further inhibiting their maturation. Overall, the study provided evidence that both BM-MSCs and their derived exosomes have potential as therapeutic agents for psoriatic skin lesions (31) (**Table 1**).

**Table 1:** Summary of the studies on extravesicular vesicles.

Source of MSC-EVs	Disease	Doses	Model	Key observation	References
Bone marrow	Psoriasis	A dose of 1 million BM-MSCs in 1 mL phosphate buffer saline	50 albino rats	BM-MSCs and exosomes significantly ameliorated psoriasis-like skin inflammation in rats. BM-MSCs secrete various cytokines that shift T lymphocyte populations from effector T cells to regulatory T cells.	Bakr et al. (31)
Bone marrow	Psoriasis	n/a	Male Balb/C mice	MSC exosomes act through CD59 to inhibit the formation of the terminal-activated C5b-9 complement complex, which subsequently inhibits NETosis and the secretion of IL-17 by neutrophils.	Zhang et al. (32)
Human umbilical cord	Contact dermatitis	10 or 50 μg/mL hUC-MSC-derived EVs	6-week-old male BALB/c mice	hUC-*MSC*-derived EVs inhibited the proliferation of cytotoxic T cells (Tc1) and type 1 helper T cells (Th1) and promoted the generation of regulatory T cells (Tregs).	Guo et al. (33)
Bone marrow	Systemic sclerosis	A dose of BMSC-EV 15 μg/100 μL	Female C57BL/6 mice	BMSC-EV treatment led to a significant reduction in TGF-β1-positive cells, α-SMA-positive myofibroblasts, mast cells, infiltrating macrophages, and lymphocytes in the skin of SSc mice.	Jin et al. (34)
Bone marrow	Systemic sclerosis	n/a	BLM-treated and IL-33-knockout mice	BMSC-Exos inhibits the IL-33/ST2 axis leading to reduced proliferation, migration, and fibrotic gene expression in fibroblasts, ultimately mitigating skin fibrosis.	Xie et al. (35)
Bone marrow	Systemic sclerosis	UC-MSC exosomes (a dose of 1 × 10^9^ particles/mL)	Human dermal fibroblasts	Treatment with UC-MSC exosomes led to an increase in the expression of collagen I and elastin, key proteins involved in skin rejuvenation.	Kim et al. (36)
Bone marrow	Systemic sclerosis	The dose of MSCs 0.1 × 10^6^ cells per 10 g body weight	B6.Cg-Fbn1Tsk/J; *Tsk*/^+^ mice	A downregulation of mTOR pathway activation to boost osteogenic differentiation and lower adipogenic differentiation	Chen et al. (37)
Bone marrow	Graft-versus-host disease	Dose of bone marrow cells, 8 × 10^6^	10- to 12-week-old BALB/cJ^H-2d^ female mice	MSCs-exo ameliorated fibrosis in the skin, lung, and liver and decreased the clinical and pathological scores of cGVHD. In pathomechanism, MSCs-exo exhibited potent immunomodulatory effects by inhibiting IL-17-expressing pathogenic T cells and inducing IL-10-expressing regulatory cells.	Lai et al. (38)
Mesenchymal	Graft-versus-host disease	The dose was 100 μg MSC-EVs	8- to 10-week-old female BALB/c (H-2d) mice	The study observed a decrease in macrophage percentage in the skin and spleen, suggesting that MSC-EVs reduced macrophage activity. Moreover, a reduction in TGF-β and SMAD2 production, key factors involved in fibrosis, was noted in the skin of MSC-EV-treated mice.	Guo et al. (39)
Mesenchymal	Alopecia areata	Th dose: 1 mL baricitinib-loaded EVs, (86.37 μg baricitinib and 2.48 × 10^11^ EV particles)	Twenty 6-week-old female C57BL/6 mice	All treatment groups (baricitinib alone, empty EVs, and EV-B) showed enhanced hair regrowth compared to the control group in mice with induced AA. EV-B decrease an inflammation, by downregulating the expression of IFN-γ, Jak-2, Stat-1, and IL-15. Additionally, to alleviate the inflammation, EV-B treatment led to a boost in the expression of β-catenin (it is responsible for hair follicle development and the anagen phase of the hair cycle).	Tang et al. (40)

BM-MSCs, bone marrow mesenchymal stem cells; IMQ, imiquimod; TGF-β, transforming growth factor-beta; HGF, hepatocyte growth factor; PGE2, prostaglandin E2; NO, nitric oxide; IDO, indoleamine 2,3-dioxygenase; DCs, dendritic cells; hUC-MSC-derived EVs, human umbilical cord mesenchymal stem cell-derived extracellular vesicles; Tc1, cytotoxic T cells; Th1, type 1 helper T cells; Tregs, regulatory T cells; SSc, scleroderma; miRNAs, microRNAs; BM-MSC-Exos, bone marrow mesenchymal stem cell-derived exosomes; BLM, bleomycin; TGF-β1, potent fibrosis-inducing growth factor; HDFs, human dermal fibroblasts; EM, effector memory; EMRA, effector memory RA; FASL, FAS ligand; TERT, telomerase reverse transcriptase; BRG1, bromodomain-containing protein 1; MSCT, mesenchymal stem cell transplantation; IF, immunofluorescence; MSCs-exo, mesenchymal stem cell-derived exosomes; cGVHD, chronic graft-versus-host disease; AA, alopecia areata.

Zhang et al. (32) investigated how topically applied EVs (i.e., exosomes) alleviate psoriasis-associated inflammation. MSC-derived EVs were administered intraperitoneally or topically applied in a mouse model of imiquimod-induced psoriasis. Reduced levels of IL-17 and C5b-9 were demonstrated in psoriatic skin treated with exosomes. Most likely, MSC-derived exosomes act through CD59 to inhibit the formation of the terminal-activated C5b-9 complement complex, which subsequently inhibits NETosis (neutrophil extracellular trap formation) and the secretion of IL-17 by neutrophils. In conclusion, this type of treatment may lead to the mitigation of the spread and amplification of inflammatory signals within psoriatic skin lesions (32) (**Table 2**).

**Table 2:** Summary of the studies on mesenchymal cells in different skin diseases.

Source of mesenchymal stem cells	Disease	Dose	Model	Key observation	References
Bone marrow	Atopic dermatitis	A dose of 2 × 10^5^ cells	Balb/c mice	BM-MSCs inhibited the proliferation and cytokine production of T cells by downregulating transcription factors such as T-bet, GATA-3, and c-Maf. BM-MSCs into ovalbumin-induced AD mice led to a significant reduction in cell infiltration in skin lesions, decreased serum IgE levels, and suppressed IL-4 expression in both lymph nodes and skin.	Na et al. (41)
Bone marrow	Atopic dermatitis	A dose of 1.0 × 10^6^ cells/kg	Five patients with atopic dermatitis	Changes were observed in certain cytokines, including a decrease in CCL-17, IL-13, and IL-22 and an increase in IL-17 in AD patients with sustained clinical response.	Shin et al. (42)
Bone marrow	Atopic dermatitis	The dose of BM-MSCs was 80 μL/day for 10 days.	BALB/c mice with AD lesions	Cell-based therapy reduced the RNA expression levels of inflammatory cytokines in the AD skin, suppressed the serum IgE levels, and decreased the proinflammatory cytokines like IL-1b, IL-4, IL-6, IL-10, and IL-13.	Ryu et al. (43)
Tonsil-derived-mesenchymal cells	Atopic dermatitis	The dose of TMSC was 2 × 10^4^.	C57BL/6J mice with AD	TMSC treatment inhibited T-cell-mediated inflammatory responses by reducing the levels of IL-6, IL-1β, TNF-α, IL-4, and B-cell-mediated serum IgE in mice with AD.	Jung et al. (44)
Dental follicle mesenchymal stem cells	Atopic dermatitis	The dose of DF-MSCs was 5 × 10^4^.	9 patients with AD and 6 patients with psoriasis	DF-MSCs alleviate inflammation by reducing T cell apoptosis, expanding Tregs, and stabilizing cytokines	Zibandeh et al. (45)
Human umbilical cord blood	Atopic dermatitis	The dose of hUCB-MSCs was 2 × 10^6^ cells/200 μL	NC/Nga mice with AD	Activated hUCB-MSCs produce TGF-β1, which plays a crucial role in reducing mast cell degranulation by downregulating FcϵRI expression.	Kim et al. (46)
Human umbilical cord blood	Atopic dermatitis	The dose of hUCB-MSCs was 1 × 10^6^.	NC/Nga mice with AD	hUCB-MSCs more effectively suppress the activation of mast cells and B lymphocyte.	Lee et al. (4)
Human umbilical cord blood	Atopic dermatitis	Two doses of hUCB-MSCs: the higher dose of 5.0 × 10^7^ cells and the lower dose of 2.5 × 10^7^ cells.	Adult patients with moderate-to-severe AD	The serum IgE levels and blood eosinophils were downregulated by the treatment.	Kim et al. (47)
Human umbilical cord blood	Atopic dermatitis	The dose of hUCB-MSCs was 2 × 10^6^.	NC/Nga mice with AD The dose of hUCB-MSCs was 2 × 10^6^.	Pimecrolimus also impaired the immunomodulatory activity of hUCB-MSCs, including their ability to inhibit Th2 cell differentiation and mast cell activation.	Shin et al. (48)
Bone marrow	Systemic sclerosis	The dose of BM-MSCs was 6 × 10^5^ cells/cm².	Patients with SSc and SSc murine	The human counterpart of murine IL-10-producing B cells was significantly increased after an injection of alloBM-MSCs in severe SSc patients, either in all recipients or in patients with clinical improvement. Moreover, it was described as the increase of CD24^hi^CD27^pos^ memory B-cell frequency and the increase of *IL10* expression by B cells.	Loisel et al. (49)
Bone marrow	Systemic sclerosis	The dose of BMMSCs was 0.02 × 10^6^ cells.	C57BL/6J, B6.129S-*Terttm1Yjc*/J (*TERT* ^−^ */* ^−^), B6.Cg-*Fbn1Tsk*/J (Tsk/^+^), and B6Smn – mice	Treatment with BM-MSCs expressing telomerase led to a significant reduction in skin hypodermal thickness in SSc mice. Reintroduction of telomerase activity through TERT transfection restored the immunomodulatory functions of TERT−/− BM-MSCs.	Chen et al. (50)
Human umbilical cord blood	Systemic lupus erythematosus	n/a	Patients suffering from SLE	UC-MSC infusion led to a significant decline in disease activity as measured by SLEDAI and BILAG scores. Anti-double-stranded DNA antibody and antinuclear antibody quantities were lowered after MSCT infusion.	Wang et al. (51)

ε-GLT, ϵ-globin gene-linked lymphocyte activator; ε-PST, ϵ-protein synthesis; EASI, Eczema Area and Severity Index; M-MSCs, mesenchymal stem cells derived from menstrual blood; MCMC, media concentrate; TMSCs, tonsil-derived mesenchymal stem cells; DNFB, 2,4-dinitrofluorobenzene; DF-MSCs, dental follicle mesenchymal stem cells; IDO, indoleamine 2,3 dioxygenase; hUCB-MSCs, human umbilical cord blood-derived mesenchymal stem cells; MDP, muramyl dipeptide; MC, mast cell; SCORAD, SCORing for Atopic Dermatitis; Th2, type 2 helper T; NFAT3, nuclear factor of activated T cells; anti-dsDNA, anti-double-stranded DNA antibody; ANA, antinuclear antibody; SLEDAI, Systemic Lupus Erythematosus Disease Activity Index; BILAG, British Isles Lupus Assessment Group.

#### 2.1.2 Contact dermatitis

Guo et al. (33) presented the potential therapeutic role of human umbilical cord mesenchymal stem cell-derived extracellular vesicles (hUC-MSC-derived EVs) in preventing contact hypersensitivity (CHS). hUC-MSC-derived EVs inhibited the proliferation of cytotoxic T cells (Tc1) and type 1 helper T cells (Th1), both of which play crucial roles in inflammation. Additionally, hUC-MSC-derived EVs promoted the generation of regulatory T cells (Tregs), which have immunosuppressive properties. hUC-MSC-derived EVs reduced TNF-α and IFN-γ, two key pro-inflammatory cytokines, and increased the production of IL-10, an anti-inflammatory cytokine (33). Collectively, these changes downregulate inflammatory responses such as macrophage activation and recruitment of additional immune cells to the site of inflammation and thereby ameliorate contact dermatitis (**Table 1**).

UC-MSC-EVs are often observed to outperform BM-MSC-EVs in contact dermatitis due to their enriched anti-inflammatory cargo (miRNAs, proteins, and lipids), stronger suppression of Th1/Th17 responses, enhanced skin repair capabilities, and biological advantages (younger cell source and easier to obtain and culture, leading to a higher yield of EVs and lower immunogenicity). These factors caused UC-MSC-EVs to be more effective at decreasing inflammation, restoring the skin barrier, and promoting resolution in CD models. Although UC-MSC-EVs appear to be more effective in treating contact dermatitis than BM-MSC-EVs, they are not effective in treating psoriasis, likely due to differences in the inflammatory environment and the specific mechanisms involved in each disease. In psoriasis, while MSC-EVs also play a role in immunomodulation, the disease’s complex pathogenesis and the involvement of specific pathways like the NF-κB signaling pathway, which can be targeted by engineered MSC-EVs, might explain why certain engineered EVs show better results.

#### 2.1.3 Systemic sclerosis

Jin et al. (34) demonstrated the potential of BM-MSC-EVs as a therapeutic approach for skin dysfunction in scleroderma (SSc). BM-MSC-EVs showed a similar efficacy to cell-based therapy in treating SSc but with fewer regulatory requirements, making them a more suitable therapeutic option. The therapeutic effects of BM-MSC-EVs were primarily attributed to the microRNAs (miRNAs) they carried, including miR-21a, miR-143, miR-27b, miR-29a, and let-7. These miRNAs are known to have immunomodulatory and anti-fibrotic effects—for instance, the let-7 family of microRNAs plays a role in regulating immune responses by modulating the production of pro-inflammatory cytokines and receptors. Specifically, let-7 inhibits the production of cytokines like IL-8 and receptors like IL1r1 and IL23r, which are involved in Th17 cell differentiation and regulate NKT cell function. Additionally, the miRNAs in BM-MSC-EVs were involved in regulating the proliferation and differentiation of multiple cell types and various EV-related biological processes. Precisely, BM-MSC-EV treatment led to a significant reduction in TGF-β1-positive cells, α-SMA-positive myofibroblasts, mast cells, infiltrating macrophages, and lymphocytes in the skin of SSc mice. Immunohistochemical analysis showed that BM-MSC-EV treatment significantly reduced the infiltration of inflammatory cells, such as F4/80+ macrophages and CD4+/CD8+ lymphocytes, in a mouse model of SSc. Moreover, BM-MSC-EVs decreased the mRNA levels of inflammatory cytokines: IL6 and TNF-α in SSc mice. To sum up, the fact that BM-MSC-derived EVs can achieve similar therapeutic outcomes to the cells themselves, while offering a more manageable, safe, and regulatory profile, strongly enhances their development as a potential next-generation therapy for the debilitating skin manifestations of SSc (34) (**Table 1**).

Xie et al. (35) investigated the mechanism by which bone marrow mesenchymal stem cell-derived exosomes (BM-MSC-Exos) alleviate skin fibrosis. The researchers hypothesized that miR-214, carried within exosomes released by stem cells (BM-MSCs), plays a crucial role in the pathogenesis of SSc. A mouse model of skin fibrosis was induced using bleomycin (BLM), a drug known to cause lung fibrosis. Mice were treated with BM-MSC-Exos to investigate their therapeutic potential in reducing fibrosis. Samples from SSc patients were used to measure the levels of miR-214, IL-33, and ST2 (a receptor for IL-33). miRs have been shown to post-transcriptionally regulate gene expression, and miR-214 can inhibit SSc-related fibrosis. The researchers found that BM-MSC-Exos deliver miR-214, which targets IL-33 and blocks the IL-33/ST2 axis. When BM-MSC-Exos deliver a miR-214 inhibitor, it leads to increased proliferation, migration, and expression of fibrotic genes in fibroblasts that have been stimulated with TGF-β1. This indicates that miR-214 normally suppresses these fibrotic processes. Subsequently, IL-33, which acts via its receptor ST2, also induces migration, proliferation, and fibrotic gene expression in fibroblasts, mirroring the effects observed after miR-214 inhibition. This further supports the profibrotic role of the IL-33/ST2 axis. In mice treated with bleomycin (BLM) to induce skin fibrosis, knocking out IL-33 inhibited the development of skin fibrosis. Importantly, BM-MSC-Exos delivering miR-214 have been shown to ameliorate skin fibrosis in BLM-treated mice. This highlights the therapeutic potential of BM-MSC-Exos carrying miR-214 to counter fibrosis by targeting this specific pathway. Summing up, this research emphasizes the rationale for developing BMSC-derived EVs as a cell-free therapy for SSc (35) (**Table 1**).

Kim et al. (36) reported that human umbilical cord blood-derived mesenchymal stem cells exosomes can be essential for skin rejuvenation. Exosomes may penetrate the outermost layer of the epidermis within 3 h and gradually reach deeper layers. It was shown that treatment with hUC-MSC-derived exosomes (at the dose of 1 × 10^9^ particles/mL) led to an increase in the expression of collagen I and elastin, key proteins involved in skin rejuvenation. Moreover, *in vitro* experiments showed that exosomes can integrate into human dermal fibroblasts (HDFs) and promote their migration and collagen synthesis. The study found that UC-MSC exosomes were more potent than cell-based therapy in enhancing collagen and elastin expression. Overall, these results present a compelling case for the incorporation of UC-MSC exosomes into products aimed at skin rejuvenation and potentially for therapeutic applications in various skin conditions (36) (**Table 1**).

Chen et al. (37) observed in mice with SSc that mesenchymal stem cell transplantation (MSCT) led to a reduction in skin thickness. Immunofluorescence (IF) staining revealed that CD63, a marker of exosomes, co-localized with CD105, a marker of MSC, in both the femur and skin. This suggests that MSCs (at the dose of 0.1 × 10^6^ cells per 10 g of body weight) may release exosomes that contribute to their therapeutic effects. Beyond improving skin thickness, MSCT also rescued osteoporosis and autoimmune phenotypes in Tsk/+ mice. An inhibition of IL4Rα expression downregulated mTOR pathway activation to boost osteogenic differentiation and lower adipogenic differentiation. In addition, administration of MSC together with Ad-miR-151, a microRNA known to regulate fibrosis, led to a reduction in skin hypodermal thickness. This study shows an optimistic way of treatment with the MSCT in a mouse model of SSc (37) (**Table 1**).

#### 2.1.4 Graft-versus-host disease

Lai et al. (38) reported the potential of mesenchymal stem cell-derived exosomes (MSCs-exo) as a promising therapeutic approach for chronic graft-versus-host disease (cGVHD). It has been demonstrated that MSCs-exo effectively prolonged the survival of mice with cGVHD and reduced the severity of clinical and pathological manifestations. Furthermore, MSCs-exo ameliorated fibrosis in the skin, lung, and liver and decreased the clinical and pathological scores of cGVHD. It is postulated that MSCs-exo exhibited potent immunomodulatory effects by inhibiting IL-17-expressing pathogenic T cells and inducing IL-10-expressing regulatory cells. Precisely, MSCs-exo treatment significantly reduced the expression of key Th17-related pro-inflammatory cytokines, including IL-17A, IL-21, IL-22, and IL-2. Moreover, MSCs-exo treatment reduced the percentage of activated CD4+ T cells (CD4+, CD44+) in cGVHD mice compared to the control groups. MSCs-exo treatment also significantly decreased the expression of CCR6, a chemokine receptor involved in the recruitment of Th17 cells, which is implicated in cGVHD pathogenesis. Summing up, based on the above-mentioned data, this treatment with MSCs-exo could offer a new therapeutic avenue for chronic graft-versus-host disease cGVHD (38) (**Table 1**).

Guo et al. (39) investigated the therapeutic potential of MSC-EVs in sclerodermatous cGVHD. They found a decrease in macrophage percentage in the skin and spleen, suggesting that the secretome-based therapy (at the dose of 100 μg MSC-EVs every 5 days) reduced the macrophage activity. Moreover, a reduction in TGF-β and SMAD2 production, key factors involved in fibrosis, was noted in the skin of MSC-EV-treated mice. MSC-EVs were found to decrease the infiltration of macrophages, particularly the pro-inflammatory CD11b+F4/80+ subset. In addition, MSC-EVs appeared to interfere with the interaction between TFH/GC B cells and also reduced the ratio of BAFF to B cells. By suppressing macrophage activation and B cell responses, MSC-EVs may help to alleviate the inflammatory processes associated with cGVHD. According to this study, MSC-EVs possess multifaceted immunomodulatory capabilities and could be used as a fibrosis alleviator (39) (**Table 1**).

#### 2.1.5 Alopecia areata

Tang et al. (40) explored a new approach to treat alopecia areata (AA). In this study, EVs derived from mesenchymal stem cells were loaded with baricitinib (EV-B). Baricitinib is a known inhibitor of the JAK-STAT pathway, which is implicated in the inflammation associated with AA. The authors found out that all treatment groups (baricitinib alone, empty EVs, and EV-B) showed enhanced hair regrowth compared to the control group in mice with induced AA. Importantly, the EV-B group displayed the most significant improvement, with complete hair coverage in the treated area by day 20. The superior efficacy of EV-B was likely due to the improved delivery of baricitinib by the EVs and a potential synergistic effect between the drug and the EVs themselves. EV-B downregulated the expression of IFN-γ, Jak-2, Stat-1, and IL-15, all of which are essential components of the JAK-STAT signaling pathway. EV-B further demonstrated a more significant reduction in the expression of Jak-2, Stat-1, and IL-15 compared with baricitinib alone. Additionally, to alleviate inflammation, EV-B treatment increased the expression of β-catenin, a key signaling molecule in the Wnt/β-catenin pathway, which plays a crucial role in hair follicle development and the anagen phase of the hair cycle. In this case, β-catenin can be considered a marker of hair follicles in the anagen phase. In summary, EV-B promoted hair growth in a mouse model of AA by inhibiting inflammation through the downregulation of the JAK-STAT pathway and promoting hair follicle regeneration through the upregulation of the Wnt/β-catenin pathway. The use of EVs seems to be aimed at enhanced drug delivery and potentially contributes to the therapeutic effect. In summary, this type of modality is a more effective treatment strategy for AA by aiming at both the inflammatory and regenerative sides of this hair condition (40) (**Table 1**).

### 2.2 Mesenchymal stem cells

#### 2.2.1 Atopic dermatitis

Na et al. (41) highlighted the therapeutic potential of BM-MSCs in treating AD. BM-MSCs effectively suppressed the activation of both T and B cells in the atopic skin of mice. Precisely, BM-MSCs inhibited the proliferation and cytokine production of T cells by downregulating transcription factors such as T-bet, GATA-3, and c-Maf. In addition, BM-MSCs were found to significantly decrease the levels of ϵ-globin gene-linked lymphocyte activator (ε-GLT) and ϵ-protein synthesis (ε-PST), two key genes involved in IgE production. In consequence, BM-MSCs suppressed the production of immunoglobulin E (IgE) by downregulating AID and BLIMP-1, key regulators of isotype class switching and B-cell differentiation. Moreover, an intravenous injection of BM-MSCs into ovalbumin-induced AD mice led to a significant reduction in cell infiltration in skin lesions, decreased serum IgE levels, and suppressed IL-4 expression in both lymph nodes and skin. BM-MSCs migrated to the skin lesions and draining lymph nodes, indicating their ability to target the affected areas. This study presents a possibility of developing a new therapeutic strategy for AD (41) (**Table 2**).

Shin et al. (42) demonstrated the efficacy and safety of multiple doses of allogeneic BM-MSC in adult patients with moderate to severe AD that was refractory to conventional treatments. The primary outcome measures, such as Eczema Area and Severity Index (EASI), showed significant improvement at 16 weeks, with 80% of patients achieving EASI-50 after one or two treatment cycles. Long-term follow-up revealed no serious side effects and maintained clinical response in some patients for over 84 weeks. Additionally, changes in certain cytokine levels were observed, including a decrease in CCL-17, IL-13, and IL-22 and an increase in IL-17 in patients with sustained clinical response. Both the first patient and the second one achieved a significant reduction in AD symptoms, as measured by the EASI-50 score, after the first cycle of treatment. The therapeutic effects of these compounds were sustained for a prolonged period, demonstrating long-term efficacy. Patients who responded well to the treatment had relatively higher levels of IL-17 in their blood compared to other patients (42) (**Table 2**). Despite promising results, it should be emphasized that the above-mentioned study was conducted on a small scale (*n* = 5), which significantly limits its reliability and the possibility of drawing conclusions. Large-scale studies are needed to confirm the effectiveness of the therapy.

Ryu et al. (43) compared the immunoregulatory functions of mesenchymal stem cells derived from menstrual blood (M-MSCs) and BM-MSCs. They found that M-MSCs, administered at a dose of 80 μL/day for 10 days, exhibited superior immunoregulatory properties compared to BM-MSCs. Further investigation focused on M-MSC conditioned media concentrate (MCMC) in mice with AD lesions. MCMC significantly reduced the RNA expression levels of inflammatory cytokines in the skin, suppressed the serum IgE levels, and decreased proinflammatory cytokines like IL-1b, IL-4, IL-6, and IL-13. The histopathological analysis also revealed a significant improvement in skin lesions. These findings suggest that secretome from M-MSCs hold promise for effectively treating AD-related inflammatory lesions and highlight the potential of M-MSCs as a promising approach for next-generation AD therapy (43) (**Table 2**).

Jung et al. (44) investigated the therapeutic effects of tonsil-derived mesenchymal stem cells (TMSCs) in a mouse model of AD induced by 2,4-dinitrofluorobenzene (DNFB). A subcutaneous injection of TMSCs (2×10^4^) significantly improved the inflammatory symptoms in the AD mice, demonstrating therapeutic rather than protective effects. TMSC treatment inhibited T-cell-mediated inflammatory responses by reducing the levels of IL-6, IL-1β, TNF-α, IL-4, and B-cell-mediated serum IgE. Additionally, TMSCs enhanced the anti-inflammatory cytokine TGF-β. Both *in vitro* and *in vivo* findings suggest that TMSC treatment effectively improved the inflammatory skin lesions in the DNFB-induced AD mice model through its immunomodulatory effects. TMSCs demonstrated the ability to inhibit T-cell- and B-cell-mediated responses while promoting anti-inflammatory responses. Based on this research, TMSCs’ therapeutic benefits in the context of AD are primarily due to their immunomodulatory effects (44) (**Table 2**).

Zibandeh et al. (45) observed a reduction in the frequency of Fas, FasL, and TNFR II in T cells, suggesting that dental follicle mesenchymal stem cells (DF-MSCs) actively reduce the signaling pathways that promote T cell apoptosis. While reducing T cell apoptosis may initially seem counterintuitive to suppress a hyperactive immune response, in the context of a chronic inflammatory state such as AD, inappropriate T cell death or survival may contribute to the pathology. Tregs are critical for maintaining immune tolerance and suppressing excessive or erroneous immune responses. Their increased frequency signifies an enhanced ability of the immune system to self-regulate and dampen inflammatory processes, which is highly beneficial in autoimmune or allergic conditions such as AD. A more balanced T-cell turnover or specific modulation of apoptotic pathways may be beneficial. In addition, the authors observed a promotion of the frequency of regulatory T cells (Treg), which are critical for maintaining immune tolerance and suppressing excessive or erroneous immune responses (45).

Kim et al. (46) suggested that NOD2-activated human umbilical cord blood-derived mesenchymal stem cells (hUCB-MSCs) can be a promising therapeutic approach for AD. Precisely, stimulating the NOD2 receptor on hUCB-MSCs (2 × 10^6^ cells/200 μL PBS) with a specific ligand, muramyl dipeptide (MDP), enhanced their therapeutic efficacy. In addition, activated hUCB-MSCs produce TGF-β1, which plays a crucial role in reducing mast cell degranulation by downregulating FcϵRI expression. The authors suggested that PGE2 secreted by hUCB-MSCs plays a crucial role in suppressing mast cell degranulation. PGE2 exerts its inhibitory effect by binding to EP2 and EP4 receptors on mast cells. While cell-to-cell contact between hUCB-MSCs and mast cells can enhance the inhibitory effect, PGE2 alone is sufficient to inhibit degranulation. What is more, the subcutaneous administration of MDP-stimulated MSCs significantly reduces scratching behavior, lymphocyte infiltration, and eosinophil infiltration in AD mice, suggesting their ability to modulate the immune response. This data powerfully summarizes the therapeutic impact of hUCB-MSCs in the treatment course of AD (46) (**Table 2**).

Lee et al. (52) observed that pretreating hUCB-MSCs with mast cell (MC) granules can enhance their therapeutic efficacy for AD treatment. Pre-exposure of hUCB-MSCs to MC granules enhanced their ability to resolve allergic immune reactions and accelerate tissue regeneration. The priming hUCB-MSCs with mast cells enhanced their therapeutic potential for wound healing by 38% compared to CM from naive hUCB-MSCs. Treatment with MC granule-primed hUCB-MSCs (likely in an *in vivo* wound model) led to several beneficial histological alterations like suppression of granulation, suppression of immune cell infiltration, and accelerated re-epithelialization. Moreover, pretreated hUCB-MSCs more effectively suppress the activation of mast cells and B lymphocytes, both key players in AD pathogenesis. Histamine released from MC granules upregulates the COX-2 signaling pathway in hUCB-MSCs, contributing to the suppression of the allergic immune response. Interestingly, MC granule priming also enhances the wound healing ability of hUCB-MSCs. MC-primed UCB-MSCs were more effective in suppressing B cell proliferation and maturation compared to naïve UCB-MSCs. In conclusion, UCB-MSCs could serve as a huge enhancement for AD therapy (52) (**Table 2**).

Kim et al. (47) revealed that the hUCB-MSCs were well tolerated and effective in reducing the symptoms of AD, including eczema area and severity, itching, and inflammation. Specifically, the high-dose group (5.0 × 10^7^ cells) showed a greater improvement in eczema index and severity index compared to the low-dose group (2.5 × 10^7^ cells). The high-dose group had substantial reductions in AD symptoms, including a 51% decrease in lesion intensity, a 58% decrease in pruritus, and a 65% decrease in insomnia at week 12. The overall SCORing for Atopic Dermatitis (SCORAD) score, a measure of AD severity, decreased by 50%, and 45% of the patients achieved a SCORAD-50 response, indicating a significant clinical improvement. A significant decrease in blood eosinophil count was observed at weeks 8 and 12 in the high-dose hUCB-MSC-treated group compared to baseline levels, suggesting that hUCB-MSCs may have a beneficial effect on the immune system in AD (47) (**Table 2**).

Shin et al. (48) demonstrated that the coadministration of pimecrolimus with hUCB-MSCs affect the therapeutic efficacy of hUCB-MSCs in AD. hUCB-MSCs exert their immunomodulatory effects through the secretion of various soluble factors, including TGF-beta, HGF, IL-6, IL-10, NO, and PGE2. PGE2 is a particularly important factor in modulating the immune response in AD. It can regulate the activity of various immune cells, such as T cells, monocytes, B cells, and NK cells. The hUCB-MSCs were used at a dose of 2 × 10^6^ in a murine AD model. Importantly, pimecrolimus (the dose was 100 ng/mL) disrupted the therapeutic effects of hUCB-MSCs. Pimecrolimus also impaired the immunomodulatory activity of hUCB-MSCs, including their ability to inhibit type 2 helper T (Th2) cell differentiation and mast cell activation. The drug decreased the production of PGE2, a crucial immunomodulatory factor in hUCB-MSCs. Additionally, pimecrolimus downregulated the COX2–PGE2 axis by inhibiting the nuclear translocation of the nuclear factor of activated T cells (NFAT3). These findings suggest that the therapeutic efficacy of hUCB-MSCs in AD can be influenced by co-administration with certain drugs and emphasize the need for further research to optimize hUCB-MSC therapy for AD patients (48) (**Table 2**).

#### 2.2.2 Systemic sclerosis

Loisel et al. (49) studied the impact of a single injection of BM-MSCs on B-cell phenotypic, transcriptomic, and functional profiles in SSc patients, with a shift from profibrotic to regulatory B cells in clinical responders. These data altogether confirm the activation of cytotoxic T cells, unlike CD4^pos^ T cells, in SSc patients receiving alloBM-MSCs, independently from the observed clinical response. A transient increase in CD8+ T-cell frequency and count was observed at the first month post-treatment, and a decrease in naive CD8+ T cells was noted at the third month. The upregulation of HLA-DR on both effector memory (EM) and effector memory RA (EMRA) CD8+ T cells was observed early on. No significant variations in the frequency of CD4+ T-cell subsets or HLA-DR expression were observed, except for a transient decrease in EM CD4+ T cells at M1. The observed T-cell changes were independent of the clinical response to alloBM-MSC infusion. Overall, two Breg subsets, considered as the human counterpart of murine IL-10-producing B cells, were significantly increased after the injection of alloBM-MSCs in severe SSc patients, either in all recipients or in patients with clinical improvement. In addition, the increase of CD24^hi^CD27^pos^ memory B-cell frequency and the increase of *IL10* expression by B cells, as associated with the observed clinical response to alloBM-MSCs, highlight Breg as key targets of MSCs in SSc. This data serves as a significant contribution to the understanding of BM-MSC biology and their therapeutic potential in complex autoimmune conditions (49) (**Table 2**).

Chen et al. (50) provided insights into the role of telomerase in the immunomodulatory functions of BM-MSCs and their potential therapeutic application for SSc. BM-MSCs lacking telomerase activity (TERT−/−) lost their ability to inhibit T cells and ameliorate SSc disease phenotype in mice. Reintroduction of telomerase activity through TERT transfection restored the immunomodulatory functions of TERT−/− BM-MSCs. TERT, in combination with β-catenin and BRG1, formed a transcriptional complex that bound to the FAS ligand (FASL) promoter. The transcriptional complex upregulated FASL expression and enhanced the immunomodulatory function of BM-MSCs. As well as that, the study demonstrated a positive correlation between telomerase activity and FASL expression. The upregulation of FasL is mediated by the Wnt/β-catenin signaling pathway. A complex formed by telomerase reverse transcriptase (TERT), β-catenin, and bromodomain-containing protein 1 (BRG1) directly binds to the FASL promoter to enhance its transcriptional activity. By targeting the TERT/β-catenin/BRG1 complex, it may be possible to modulate FasL expression and immune responses. Interestingly, treatment with BM-MSCs expressing telomerase led to a significant reduction in skin hypodermal thickness in SSc mice, comparable to the control group. This study furthermore provides a promising new direction to develop more effective BM-MSC-based therapy for patients suffering from SSc (50) (**Table 2**).

#### 2.2.3 Systemic lupus erythematosus

Wang et al. (51) found hUCB-MSCs as a safe and effective treatment option for severe and refractory SLE. hUCB-MSCs were intravenously administered to patients with SLE and a significant improvement in disease activity, as well as amelioration of cutaneous symptoms, was observed. Importantly, hUCB-MSCs were well tolerated by patients with SLE, with no serious adverse events reported. Moreover, it was shown that, in serum, an anti-double-stranded DNA antibody (anti-dsDNA) and antinuclear antibody (ANA) level were lowered after MSC transplantation (MSCT), with statistically significant differences spotted at the follow-up performed in 6 and 12 months. hUCB-MSC infusion led to a significant decline in disease activity, as measured by Systemic Lupus Erythematosus Disease Activity Index (SLEDAI) and British Isles Lupus Assessment Group (BILAG) scores (51) (**Table 2**).

## 3 MSCs and MSC-derived EVs’ efficacy across different skin diseases

BM-MSC-derived EVs hold significant promise for the treatment of cutaneous manifestations of scleroderma. They elicit therapeutic effects similar to those achieved by the cells themselves while offering improved safety and regulatory benefits. This makes them a compelling alternative to cell-based therapies for SSc, especially considering the challenges associated with cell-based approaches (34). Furthermore, preclinical studies have shown that MSC-EVs can alleviate GvHD symptoms, reduce mortality, and improve overall survival in animal models (38, 39). Similarly, the use of MSC-derived EVs in drug delivery (e.g., baricitinib) for alopecia areata (AA) shows promise to improve treatment outcomes. EVs can act as carriers for therapeutic agents, enhancing their delivery to targeted areas and potentially improving the efficacy. This targeted approach, combined with the ability of EVs to modulate inflammation and promote hair regeneration, suggests a more effective treatment strategy for AA than conventional methods (40).

Clinical studies have shown the effectiveness of BM-MSC transplantation in the reduction of AD symptoms, including Eczema Area and Severity Index (EASI) scores, Investigator’s Global Assessment (IGA) scores, and pruritus (itching). Additionally, BM-MSCs can modulate the immune system by influencing the balance of T helper (Th) cell subsets (Th1/Th17) and reducing inflammatory factors (42). Furthermore, menstrual blood-derived mesenchymal stem cells (M-MSCs) have been shown to be more effective in treating AD than BM-MSCs due to their higher proliferation rate, easier and less controversial isolation, and potential for improved homing and engraftment at injury sites (43). The immunoregulatory potential of dental follicle-derived mesenchymal stem cells in AD has been confirmed. Specifically, DF-MSCs have demonstrated the ability to reduce inflammatory cytokine levels and promote the frequency of regulatory T cells (Tregs), which play a crucial role in suppressing immune response (45). Finally, human umbilical cord blood-derived mesenchymal stem cells (UCB-MSCs) are well tolerated and effective in reducing the symptoms of atopic dermatitis, including eczema area and severity, itching, and inflammation (47). While preclinical and small-scale clinical studies are promising, large clinical trials are needed to confirm the safety and efficacy of MSC therapy for treating skin diseases in humans.

## 4 Safety and adverse reactions and risks

Since the isolation of MSCs often requires the use of invasive procedures, approaches that only require an *in vitro* culture of MSCs and use of the released product (i.e., EVs) offer as alternatives to MSCs. MSC-EVs cannot self-replicate, which is a key advantage over native MSCs when considering cell-based therapies. This lack of self-replication addresses concerns about uncontrolled cell division and potential tumorigenicity associated with MSCs. However, in one study, researchers found that hUC-MSC-derived EVs exhibited the potential for uncontrolled differentiation and proliferation, which may lead to undesirable long-term side effects (33). This inherent feature of hUC-MSC-derived EVs has been reported as a factor limiting their clinical application (33). On the other hand, most of the available studies confirmed the safety and efficacy of MSCs and MSCs-derived EVs in the treatment of dermatoses—for example, Shin et al. (42), Kim et al. (47), Shin et al. (48), and Wang et al. (51) did not find any serious adverse events in their long-term follow-up or clinical studies. Kim et al. (47) reported that most of the adverse events were local reactions at the injection site (induration, bruising, erythema, pain), which were transient and mild. Interestingly, the investigators also noted a rare occurrence of transient and mild skin infections or gastrointestinal disorders. Furthermore, Kim et al. (47) found that hUCB-MSCs were well tolerated in their study. Similarly, Wang et al. (51) found that hUCB-MSC transplantation was safe, well tolerated, and not associated with any adverse transplant-related events. The available studies indicate that the proposed therapy has a good safety profile, with mild and transient local reactions, but no serious adverse events were reported.

## 5 Challenges and future perspectives

Although MSC-derived EV therapy has numerous advantages, there are several drawbacks that limit the broader translational use of EVs. First of all, the methods for the isolation and purification of EVs should be standardized to ensure reproducibility. Quality control and standardization of EVs are crucial for their safe and effective use as a therapeutic agent. Ensuring consistency in Evs’ isolation, purification, characterization, and production is essential to minimize variability and maximize their therapeutic potential. Several techniques are employed to detect and isolate EVs based on their size, for example: ultrafiltration, sequential filtration, size exclusion chromatography, immunoaffinity chromatography, anion-exchange chromatography, electrophoresis, and dielectrophoresis (DEP) based techniques, commercial kits, field-flow fractionation, and hydrostatic filtration dialysis. There are three centrifugation-based protocols for isolating EVs from biological samples (e.g., cell culture media, urine, plasma, serum) based on their physical properties, i.e., differential ultracentrifugation, zonal gradient centrifugation, and isopycnic gradient centrifugation. Among them, differential ultracentrifugation is considered the “gold standard” for EVs’ isolation due to its ability to effectively separate EVs based on size and density, yielding relatively pure samples. However, its scalability for clinical use is limited for several reasons. UC requires multiple centrifugation steps, often taking 4–24 h per sample, with manual handling that increases variability and labor costs. UCs are expensive and require specialized maintenance and trained personnel, which make them impractical for high-throughput clinical settings. Additionally, UC processes one sample at a time, which limits it for large-scale clinical applications where hundreds or thousands of samples may need to be evaluated. UC typically requires large starting volumes (e.g., 10–100 mL), which may not be feasible for clinical samples like blood or urine, where the volumes are often limited. While UC can achieve high purity, it often results in low EV yields (5%–20% recovery) and can co-isolate contaminants, for instance, protein aggregates, affecting the consistency for clinical diagnostics or therapeutics. Methods like size exclusion chromatography (SEC), tangential flow filtration (TFF), or microfluidics-based approaches are being explored for clinical use. These offer higher throughput, better scalability, and compatibility with smaller sample volumes, though they may compromise on purity or require optimization (53). Nevertheless, the choice of purification method depends on various factors, such as initial sample volume, purity, and yield, downstream application, and available resources (54). Another problem is the lack of unique markers for the heterogeneous subclasses of EVs. Although tetraspanins such as CD9, CD63, and CD81 are currently most commonly used to characterize extracellular vesicles, their expression can vary considerably depending on the cell type producing the EVs. The low presence of the above-mentioned tetraspanins directly limits the efficiency of immunoaffinity isolation of EVs from cell populations that naturally express low levels of CD9, CD63, or CD81. As a result, a significant amount of EVs may be missed or inefficiently isolated, leading to incomplete or biased exosome yields. Afterwards, clinical-grade EVs should be manufactured in accordance with good manufacturing practice (GMP) and quality control (QC) standards. The QC criteria include determining the quantity, size, identity, and purity of EVs. One method to monitor vesicle purity is to determine the particle-to-protein, protein-to-lipid, or RNA-to-particle ratios. Another approach is to determine the expression of intracellular proteins such as histones, lamin A/C, GRP94, or cytochrome C since extracellular vesicles do not contain these proteins. Moreover, contaminants from the cell culture process, including antibiotics and serum, should also be monitored. It is also necessary to investigate which subpopulations of EVs have the highest therapeutic efficacy, as the composition of EVs may vary depending on the MSCs’ origin and culture conditions. Finally, the appropriate therapeutic doses and optimal route(s) of Evs’ administration should be established. EVs can be administered through various routes, including intravenous, topical, and intralesional—for example, Kim et al. showed that a dose of 5.0 × 10^7^ cells (hUCB-MSCs) administered subcutaneously resulted in a significant reduction in AD symptoms, including the area and severity of eczema, itching, and inflammation (47). However, most of the available studies are animal studies or small-scale clinical trials, which have a significant impact on the inability to draw firm conclusions. Summing up, the ideal protocol should be characterized by a low level of sample contamination, preservation of vesicle integrity, high efficiency, reproducibility, versatility, low cost, high isolation rate from a large number of samples simultaneously (ideally for no more than 1 h), availability and simplicity of the equipment, and the possibility of process automation. In general, the final outcome (EVs purity and yield) highly depends on the chosen isolation protocol, and the best method is determined by the specific goal of the further study (55).

## 6 Conclusions

This review presents the potential of different types of mesenchymal stem cells (MSCs) and their EVs as a therapeutic strategy for immune-mediated inflammatory skin diseases. EVs possess immunosuppressive and immunomodulatory properties that can alleviate inflammation. Importantly, EVs are less immunogenic than cells, minimizing adverse immune responses. Available preclinical and clinical studies have shown the safety and good tolerability of MSCs and their EVs in the treatment of dermatoses. On the other hand, mild and transient local reactions have been demonstrated, but without serious adverse events. While the initial results are promising, larger-scale clinical trials are necessary to establish long-term safety and efficacy. Further research is also needed to elucidate the precise mechanisms of action of EVs in skin diseases. Exploring combination therapies with other modalities, such as drugs or other biological agents, may enhance therapeutic outcomes. Developing personalized EV-based therapies tailored to individual patient needs may improve the treatment efficacy and reduce side effects. Another issue is the difficulty in producing large quantities of clinically applicable extracellular EVs resulting from the technical challenges associated with long-term cell culture and obtaining homogenous MSCs for EV generation. These challenges include limited EV yield in standard cell cultures, the need for efficient isolation and purification methods, and ensuring consistency in EV cargo and properties. In addition, the biological properties of MSCs are not uniform and can vary significantly depending on several factors, including their origin (tissue source), the method of culture and expansion, the age of the donor, and the method used to isolate them. These variations can lead to inconsistencies in the cellular characteristics across different batches of MSCs, which can impact their therapeutic potential and the outcome of clinical trials. In conclusion, MSCs and EV-based therapies may hold great promise for treating immune-mediated inflammatory skin diseases, but robust clinical trials are needed to confirm their safety and efficacy in humans before routine clinical use.
